# Evaluating the myocardial functions of the forgotten right ventricle in children with nephrotic syndrome utilizing traditional echocardiography, two-dimensional speckle tracking and three-dimensional echocardiography

**DOI:** 10.1186/s13052-026-02335-1

**Published:** 2026-09-19

**Authors:** Amira Hussein, Shaimaa Rakha, Asmaa Bakr, Alshaymaa Ahmed Ali, Ayman Hammad, Mona Hafez, Mai S. Korkor

**Affiliations:** 1 Pediatric Cardiology Unit, Faculty of Medicine, Mansoura University, Mansoura, Egypt; 2 Pediatric Cardiology Unit, Faculty of Medicine, Zagazig University, Zagazig, Egypt; 3 Pediatric Nephrology Unit, faculty of medicine, Mansoura University, Mansoura, Egypt

**Keywords:** Nephrotic syndrome, Right ventricle, 3D echocardiography

## Abstract

**Background:**

Primary nephrotic syndrome (NS) is associated with systemic effects that may impact cardiac function, although several studies have explored left ventricle in children with NS. Few studies investigated right ventricle (RV) in this disease. Therefore, the study aimed to provide a comprehensive evaluation of RV functions in children with primary NS using echocardiography.

**Methods:**

A case-control observational study was conducted from January 2022 to August 2024 on 40 primary NS patients with steroid-resistant nephrotic syndrome (SRNS) and steroid-sensitive nephrotic syndrome (SSNS), alongside 40 healthy controls. All participants underwent RV assessments using conventional parameters, tissue Doppler imaging (TDI), two-dimensional speckle-tracking echocardiography (2D STE), and three-dimensional echocardiography (3DE).

**Results:**

Significantly lower s’, e’ in NS patients compared to controls, especially lower in SRNS compared to SSNS. Significantly higher E/e’ and Tei index was detected in the NS group compared to controls (p = < 0.001) and more pronounced in SRNS. RV free wall strain longitudinal strain (RVFWLS) and RV four chamber longitudinal strain (RV4CSL) were significantly reduced in NS group compared to controls. 3D auto RV-derived ejection fraction (EF), TAPSE, stroke volume and septal strain were lower in NS compared to controls(p = < 0.001); however, there are no significant differences between SSNS and SRNS. Significant correlations were detected between some echocardiographic parameters and serum cholesterol, albumin, and creatinine.

**Conclusions:**

Integrative use of variable echocardiographic modalities allows detection of subclinical alterations in RV function in children with primary NS, with more affected parameters in SRNS. Therefore, regular surveillance of RV functions in primary NS should be considered.

## Background

Nephrotic syndrome (NS) is one of the most common glomerular diseases in children, characterized by heavy proteinuria, hypoalbuminemia, edema, and hyperlipidemia. While the primary concern in NS is renal involvement, systemic complications, including cardiovascular dysfunction, have become increasingly recognized. Chronic volume overload, dyslipidemia, prolonged corticosteroid use, and frequent relapses can predispose children to cardiovascular changes, including myocardial dysfunction and vascular abnormalities [1, 2]. Among cardiac concerns, right ventricular (RV) involvement remains underexplored despite the RV’s susceptibility to pressure and volume overload.

Patients with steroid-resistant nephrotic syndrome (SRNS) often experience a more aggressive course compared to those with steroid-sensitive nephrotic syndrome (SSNS). SRNS is frequently associated with persistent proteinuria, chronic kidney disease progression, and higher exposure to immunosuppressive therapies [3]. These factors may contribute to cardiovascular strain through mechanisms such as endothelial dysfunction, chronic inflammation, and altered fluid dynamics. While many studies have documented left ventricular abnormalities in NS using advanced echocardiographic modalities [4–6], limited data exist on the impact of NS subtype on RV structure and function.

Conventional echocardiographic parameters, such as tricuspid annular plane systolic excursion (TAPSE) and right ventricular fractional area change (RVFAC), are widely used for RV assessment. Still, they may fail to detect early or subtle dysfunction. Recent advances in cardiac imaging, including tissue Doppler imaging (TDI), three-dimensional echocardiography (3DE), and speckle-tracking echocardiography (STE), have improved the sensitivity for detecting subclinical myocardial abnormalities [7]. These techniques provide insights into myocardial deformation, volume changes, and systolic function beyond what standard 2D measurements offer. In particular, RV global longitudinal strain (GLS) has emerged as a reliable marker for early RV impairment in systemic and pulmonary diseases [8, 9].

To date, few studies have explored RV function in the context of nephrotic syndrome. To the best of our knowledge, this is the first study to compare SRNS and SSNS using a multimodal echocardiographic approach. This study aims to fill this knowledge gap by providing a comprehensive evaluation of RV function using advanced echocardiographic techniques in children with SRNS and SSNS, compared to healthy controls. Identifying early cardiac changes in these patients could inform monitoring strategies.

## Methods

### Study design

The study is a prospective case-control study that included 80 participants. Children with primary nephrotic syndrome (NS) were recruited between January 2022 and August 2024 from the nephrology outpatient clinic at Mansoura University Children’s Hospital, Egypt—a tertiary referral center with all pediatric subspecialties. Written informed consent for enrollment in the study was obtained from the legal guardians of all participants. The study protocol was approved by the Institutional Review Board (IRB) of the Faculty of Medicine, Mansoura University (R.24.07.2713).

### Inclusion criteria

The study included Patients diagnosed with Primary NS according to the International Study for Kidney Disease in Childhood (ISKDC) criteria [10]. Patients should be 1–18 years old who have been diagnosed at least one year prior to enrollment in the study. Moreover, all subjects included were in remission, defined as three consecutive days of trace or negative protein in urinalysis. Participants had normal serum complement C3 levels and preserved kidney function, defined as an estimated glomerular filtration rate > 90 ml/min/1.73 m² (calculated using the Schwartz formula) [11]. An age-matched healthy control group was included for comparison.

### Exclusion criteria

Patients were excluded if they had any of the following: hemodynamically significant congenital heart disease, primary cardiomyopathy, coronary artery disease, or arrhythmia. Moreover, patients with impaired renal function, secondary NS (e.g., diabetes mellitus or systemic lupus erythematosus), or any other systemic disease known to impair cardiac function were not recruited for the study. Additionally, patients with suboptimal echocardiographic image quality for RV assessment were also excluded.

### Sample size calculation

Sample size was calculated using G*power version 3.1.9.7. The calculation was based on using the mean and SD of RV EF% results of a pilot of 5 cases and 5 controls. The sample size was 31 participants per group (patients and controls), with a 5% level of significance and 90% power. The sample size will be increased in each group to enhance the study’s power.

### Basic demographic, clinical, laboratory, and therapeutic data

All enrolled children underwent comprehensive clinical data collection, including documentation of age, sex, duration of NS, response to corticosteroids, relapse frequency per year, duration of steroid therapy and the current doses, and details of other medications as mycophenolate mofetil (MMF) and ciclosporin. Anthropometric data, including weight, height, and body mass index (BMI), were obtained. Moreover, blood pressure and heart rate were recorded immediately before echocardiographic examination.

Relevant biochemical parameters were retrieved from medical records, including serum creatinine, albumin, and total cholesterol at both the initial diagnosis of NS and around cardiac evaluation.

### Echocardiographic assessment

Transthoracic echocardiograms (TTE) were conducted using the EPIQ CVx system (Philips Medical Systems, Bothell, WA, USA, 2018) equipped with an X5-1 matrix-array probe (5–1 MHz).

#### Standard 2D echocardiography with color Doppler

Two-dimensional imaging and color Doppler were acquired in accordance with the American Society of Echocardiography (ASE) guidelines [12]. It was used to exclude structural abnormalities and assess the presence of tricuspid and pulmonary valvular regurgitation. M-mode was used to exclude cases with an evident low ejection fraction (EF).

#### Pulsed-wave conventional doppler

Doppler interrogation across the tricuspid valve provided early (E) and late (A) diastolic inflow velocities. The E/A ratio was subsequently calculated to assess diastolic function.

#### Pulsed-tissue doppler imaging (TDI)

TDI was applied to the lateral tricuspid annulus from the apical 4-chamber view. Early diastolic (e′), late diastolic (a′), and systolic (s′) myocardial velocities were recorded in centimeters per second. The E/E′ ratio was calculated in addition to the RV Tei index measured as (IVCT + IVRT)/ET. The RV Tei index was used to assess combined systolic and diastolic performance. Ejection time (ET) was derived from the systolic (s′) wave. All values were averaged over three consecutive cardiac cycles.

#### Two-dimensional speckle tracking echocardiography (2D-STE)

Dedicated RV-focused apical four-chamber views were utilized for 2D-STE analysis. Longitudinal strain values of the RV free wall and interventricular septum were assessed. The primary parameters studied were RV free wall longitudinal strain (RVFWLS) and RV global 4-chamber Strain (RV4CSL), analyzed using Auto Strain RV software.

#### Three-dimensional (3D) RV functional and volumetric analysis

**Fig. 1:**
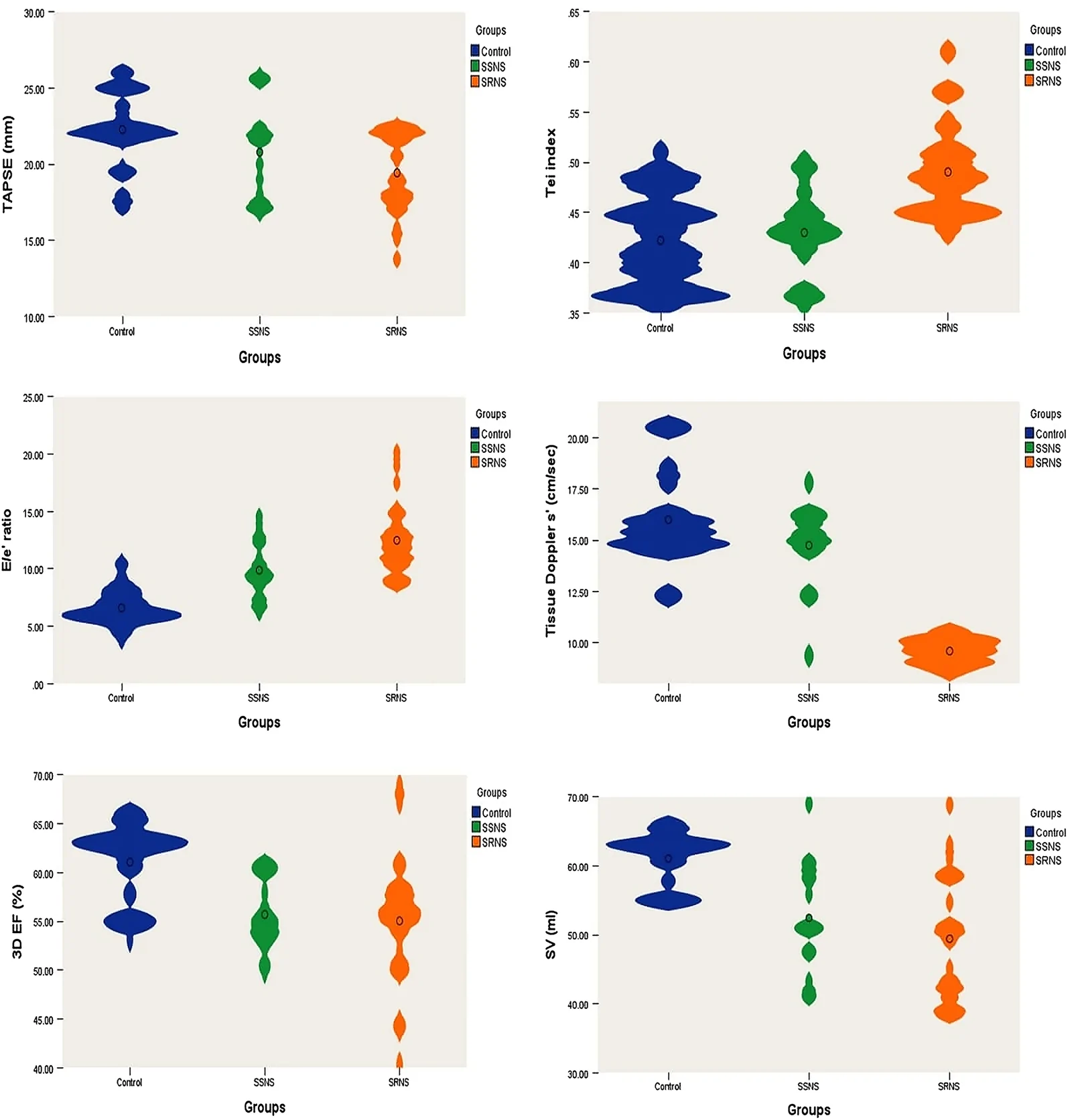
Violin plot comparing the distribution of RV Tei index, TAPSE, E/e ratio, S velocity, 3D-SV, and 3D EF in the three study groups. The width of the violin indicates the density of cases, and the black circle inside stands for the mean. 3D: three-dimensional, EF: ejection fraction, SRNS: steroid-resistant nephrotic syndrome, SSNS: steroid-sensitive nephrotic syndrome, SV: stroke volume. The black circle stands for the mean

RV-focused full-volume ECG-gated datasets were obtained using the Heart Model Acquisition (HM ACQ) mode with a frame rate of more than 20–25 fps. The data were stored and analyzed using the Dynamic HeartModel A.I. software, which automatically quantified right ventricular volumes and function using 3D speckle-tracking algorithms. The software automatically generates the following 3D-derived parameters: end-diastolic volume (EDV), end-systolic volume (ESV), stroke volume (SV), and RV ejection fraction (RVEF). Moreover, the software calculates the following 2D-derived measurements: tricuspid annular plane systolic excursion (TAPSE), RV systolic fractional area change (FAC), and RV longitudinal Strain at the septum and free wall. An example of 3D RV analysis is presented in Fig. 1.

**Fig. 2:**
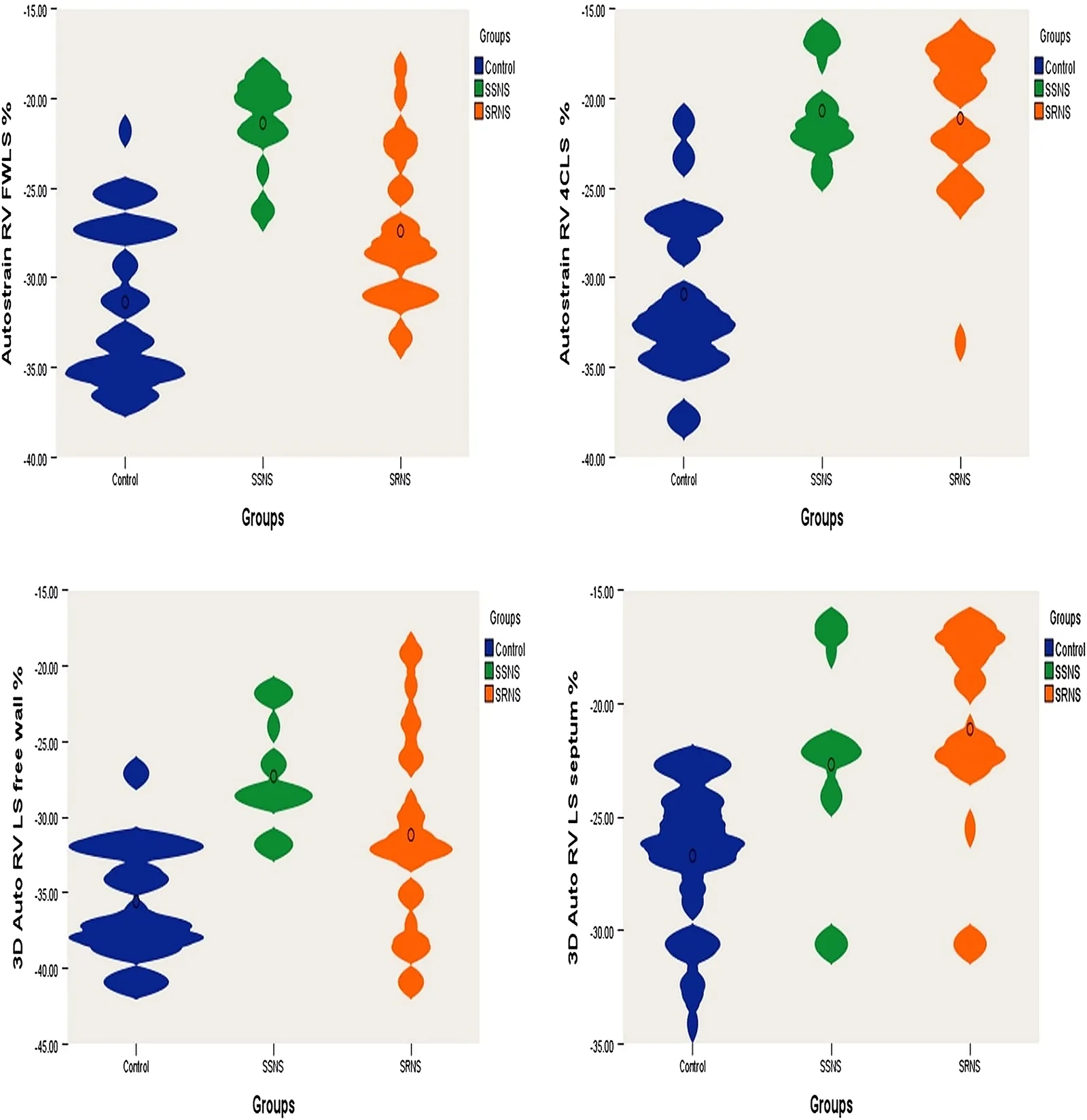
Violin plot comparing the distribution of RV 2D and 3D speckle tracking-derived strains in the three study groups. FWLS: free wall longitudinal strain, RV4CLS: right ventricular four chamber global longitudinal strain, RVLS: right ventricular longitudinal strain, SRNS: steroid resistant nephrotic syndrome, SSNS: steroid sensitive nephrotic syndrome. The violin’s width indicates the density of cases, and the black circle inside represents the mean

**Table 1:** Demographic and clinical data of the studied groups

		SSNS (*n* = 15)	SRNS (*n* = 25)	*P* value
Age (years)		11 (8–12.5)	12 (4–15.5)	0.106
Gender (Male)		14 (93.3%)	15 (60%)	0.030*
Weight (kg)		40 (25–50)	57 (16–90)	0.013*
Height (cm)		153 (126–155)	152 (96–172)	0.376
BMI (kg/m^2^)		17.1 (15.8–20.8)	23.8 (16–31.6)	< 0.001*
Surface area		1.3 (0.94–1.47)	1.57 (0.65–2.09)	0.020*
Clinical and therapeutic data	**Number of relapses/ years**	1 (1–2)	1 (0–3)	0.66
	**Number of hospitalization/ years**	0 (0–0)	0 (0–2)	0.01
	**Disease Duration in years**	6 (1–6)	3 (2.25–6.25)	0.43
	**Duration of steroid (months)**	36 (12–80)	39 (12–150)	0.11
	**Current dose of steroids (mg/m** ^**2**^ **/day)**	20 (15–30)	10 (0–45)	0.02*
	**Ciclosporin therapy**	0 (0%)	25 (100%)	< 0.001*
	Duration (months)	-	27 (6–96)	-
	Current dose (mg/kg/day)	-	3 (0–4)	-
	**MMF therapy**	0 (0%)	4 (24%)	
	Duration (months)	**-**	23 (10–48)	-
	Dose (mg/m^2^/dose)	**-**	400 (300–750)	-
	**Hypertension (on antihypertensives)**	0 (0%)	10 (40%)	0.006*
Laboratory Data	**Current serum creatinine (mg/dl)**	0.6 (0.4–0.6)	0.5 (0.5–0.6)	0.72
	**Current serum Albumin (gm/dl)**	4.5 (4.1–4.7)	4 (3.7–4.25)	0.009*
	**Quantitation of proteinuria at the time of study (A/C ratio)**	0.15 (0.1–0.2)	0.1 (0–0.9)	0.62

BMI: body mass index, SSNS: Steroid sensitive nephrotic syndrome, SRNS: steroid resistant nephrotic syndrome, ACR: albumin/creatinine ratio, MMF: mycophenolate mofetil, Data are presented as median (IQR) or number (Percentage)

*: Significant P value. Table 1 was previously reported in our study evaluating left ventricular function and is reproduced here for completeness and to facilitate interpretation of the present study [6]

**Table 2:** Comparison between the groups studied regarding conventional and Tissue Doppler RV echocardiographic parameters

Parameters		Control (*n* = 40)	SSNS (*n* = 15)	SRNS (*n* = 25)	p1	P2	P3	p4	p5
Conventional ECHO	TV E (cm/sec)	78 0.48 ± 15.4	75.15 ± 6.6	70.1 ± 10.3	0.04*	0.026*	0.42	0.019*	0.09
	TV A (cm/sec)	46.28 ± 5.23	52.61 ± 14.07	50.68 ± 7.88	0.02*	0.007*	0.017*	0.009*	0.58
	TV E/A	1.72 ± 0.4	1.5 ± 0.4	1.41 ± 0.3	0.005*	0.002*	0.14	0.001*	0.26
	TR (n, %)	6 (15%)	5 (33.3%)	12 (48%)	0.015*	0.013*	-	-	-
	PR (n, %)	3 (7.5%)	7 (46.7%)	10 (40%)	< 0.001*	< 0.001*	-	-	-
Tissue Doppler	RV s’ (cm/sec)	16 ± 2.18	14.8 ± 2.9	9.59 ± 0.6	< 0.001*	< 0.001*	0.36	< 0.001*	< 0.001*
	RV e’ (cm/sec)	12.1 ± 2.1	8.01 ± 1.9	5.8 ± 1.25	< 0.001*	< 0.001*	< 0.001*	< 0.001*	< 0.001*
	RV a’ (cm/sec)	10.16 ± 2.8	10.57 ± 1.3	11.18 ± 2.3	0.20	0.59	0.09	0.09	0.16
	E/e’	6.6 ± 1.48	9.89 ± 2.4	12.48 ± 3.1	< 0.001*	< 0.001*	< 0.001*	< 0.001*	0.008*
	RV Tei index	0.36 ± 0.05	0.40 ± 0.06	0.47 ± 0.07	< 0.001*	< 0.001*	0.06	< 0.001*	0.003*

FAC: fractional area change, PR EDV: Pulmonary regurgitation end-diastolic velocity, RVID: right ventricle internal diameter, SRNS: steroid resistant nephrotic syndrome, SSNS: steroid sensitive nephrotic syndrome, TAPSE: tricuspid annular plane systolic excursion, TR: tricuspid regurgitation, TV: tricuspid valve. Data in columns of control, SSNS, and SRNS are presented as mean ± SD. p1: Comparing the three studied groups, p2: Comparing Control and NS, p3: Comparing control and sensitive, p4: Comparing control and resistant, p5: Comparing sensitive and resistant. *: Significant

**Table 3:** Comparison between 2D-STE derived strains of the study groups

	Control (*n* = 40)	SSNS (*n* = 15)	SRNS (*n* = 25)	p1	p2	p3	p4	p5
Global Strain								
RVFWSL (%)	-31.35 ± 4.3	-21.37 ± 2.5	-27.38 ± 4.1	< 0.001*	< 0.001*	< 0.001*	< 0.001*	< 0.001*
RV4CSL (%)	-30.9 ± 4.4	-20.67 ± 2.6	-21.08 ± 4	< 0.001*	< 0.001*	< 0.001*	< 0.001*	0.72
Segmental Strain								
Base (%)	-30.2 ± 5.54	-21.5 ± 5.06	-28.5 ± 2.8	< 0.001*	0.002*	< 0.001*	0.26	0.001*
Mid (%)	-28.4 ± 1.81	-21.9 ± 5.39	-20.7 ± 4.3	< 0.001*	< 0.001*	< 0.001*	< 0.001*	0.30
Apical (%)	-28.4 ± 1.81	-22 ± 5.30	-23.28 ± 6.1	< 0.001*	< 0.001*	0.001*	< 0.001*	0.52

RVFWSL: right ventricular free wall longitudinal strain. RV4CSL: right ventricular 4-chamber longitudinal strain

SRNS: steroid resistant nephrotic syndrome, SSNS: steroid sensitive nephrotic syndrome

Data in columns of control, SSNS, and SRNS are presented as mean ± SD

p1: Comparing the three studied groups, p2: Comparing Control and NS, p3: Comparing control and sensitive

p4: Comparing control and resistant, p5: Comparing sensitive and resistant. *: Significant

**Table 4:** Comparison between three studied groups regarding 3D auto RV derived parameters

3D Auto RV	Control (*n* = 40)	SSNS (*n* = 15)	SRNS (*n* = 25)	p1	p2	p3	p4	p5
EDV (ml)	68 ± 15.9	85.6 ± 9.25	87.1 ± 9.42	< 0.001*	< 0.001*	< 0.001*	< 0.001*	0.94
EDVI (ml/m2)	58.6 ± 17.2	75.8 ± 14.5	66.7 ± 23.5	0.007*	0.02*	0.002*	0.30	0.036*
ESV (ml)	38.33 ± 8.23	58.65 ± 7.06	55.68 ± 8.57	< 0.001*	< 0.001*	< 0.001*	< 0.001*	0.36
ESVI (ml/m2)	33.23 ± 8.67	51.21 ± 9.35	44.01 ± 20.67	< 0.001*	< 0.001*	< 0.001*	0.06	0.03*
SV (ml)	61.05 ± 3.8	52.46 ± 7.9	49.43 ± 8.9	< 0.001*	< 0.001*	< 0.001*	< 0.001*	0.28
3D-EF (%)	61.85 ± 3.9	55.7 ± 3.5	55.05 ± 6.4	< 0.001*	< 0.001*	< 0.001*	< 0.001*	0.72
RVDd- base (mm)	30.2 ± 2.52	29.6 ± 2.98	38.8 ± 2.55	< 0.001*	< 0.001*	0.38	< 0.001*	< 0.001*
RVDd- mid (mm)	32.35 ± 9.7	28.7 ± 2.93	34.38 ± 2.91	< 0.001*	0.09	0.016*	< 0.001*	< 0.001*
RVLd- apex (mm)	64.71 ± 5.61	59.77 ± 3.81	67 ± 5.96	< 0.001*	0.87	0.006*	0.030*	< 0.001*
TAPSE (mm)	22.28 ± 28	20.79 ± 3.18	19.44 ± 2.63	< 0.001*	< 0.001*	0.06	< 0.001*	0.15
FAC %	54.85 ± 4.85	53.72 ± 3.54	55.05 ± 4.02	0.66	0.59	0.30	0.97	0.61
RVLS- septum %	-26.7 ± 3.17	-22.67 ± 4.86	-21.11 ± 4.39	< 0.001*	< 0.001*	< 0.001*	< 0.001*	0.30
RVLS- free wall %	-35.56 ± 3.7	-27.29 ± 3.25	-31.16 ± 6.39	< 0.001*	< 0.001*	< 0.001*	< 0.001*	0.038*

EDV: end-diastolic volume, EDVI: end-diastolic volume index, EF: Ejection fraction, ESV: End systolic volume, ESVI: End systolic volume indexed, FAC: Fractional area change, RVLS: right ventricular longitudinal strain, SV: RVDd: right ventricle dimension at end-diastole, SRNS: steroid resistant nephrotic syndrome, SSNS: steroid sensitive nephrotic syndrome, SV: Stroke volume, TAPSE; tricuspid annular plane systolic excursion, Data in columns of control, SSNS, and SRNS are presented as mean ± SD

p1: Comparing the three studied groups, p2: Comparing Control and NS, p3: Comparing control and sensitive,

p4: Comparing control and resistant, p5: Comparing sensitive and resistant. *: Significant

**Table 5:** Correlations of RV functions with Clinical, laboratory, and therapeutic characteristics of the included NS patients

	RV S’		Tei Index		FAC (%)		TAPSE (mm)			RVFWSL Autostrain RV (global)			RV4CSL Autostrain RV (Global)			3D EF (%)	
	*R*	*P*	*r*	*P*	*r*	*P*		*r*	*P*	*r*	*P*		*r*		*P*	*r*	*P*
Disease Duration	0.06	0.72	-0.07	0.68	-0.04	0.83	0.13		0.44	0.02		0.92		-0.06	0.69	0.07	0.66
Relapses (per year)	-0.05	0.78	0.09	0.60	-0.01	0.97	-0.08		0.62	-0.13	0.41		-0.01		0.95	0.06	0.73
Steroid Duration (month)	-0.25	0.12	0.14	0.39	0.05	0.74	0.03		0.84	-0.22	0.18		-0.21		0.20	-0.07	0.67
Ciclosporin Duration (for SRNS in month)	0.006	0.98	0.14	0.90	-0.09	0.69	0.13		0.54	0.12	0.57		-0.06		0.76	0.26	0.22
S. Creatinine (initial)	-0.34	0.03*	-0.03	0.84	-0.22	0.18	-0.08		0.63	-0.33	0.04*		-0.41		0.008*	0.001	0.99
S. Creatinine (latest)	-0.14	0.37	-0.07	0.66	-0.03	0.86	-0.24		0.14	-0.26	0.1		0.003		0.98	-0.45	0.004*
S. Cholesterol (initial)	0.09	0.56	0.22	0.17	-0.04	0.79	-0.008		0.96	0.16	0.32		0.23		0.15	-0.04	0.79
S. Cholesterol (latest)	-0.57	0.001*	0.45	0.004*	0.24	0.14	-0.14		0.4	-0.51	0.001*		-0.3		0.049*	-0.13	0.41
S. Albumin (Initial)	0.51	0.001*	-0.25	0.12	-0.15	0.37	-0.006		0.97	0.41	0.008*		0.16		0.32	-0.05	0.76
S. Albumin (latest)	0.41	0.008*	-0.09	0.58	-0.13	0.44	0.15		0.37	0.4	0.01*		-0.11		0.52	0.1	0.52

EF: ejection fraction; FAC: Fractional area change, GLS: global longitudinal strain, RVFWSL: right ventricular free wall longitudinal strain. RV4CSL: right ventricular 4-chamber longitudinal strain. TAPSE; tricuspid annular plane systolic excursion, 3DE: three-dimensional echocardiography. *r*: Pearson’s correlation coefficient, *: Significant

### Statistical methods

Data analysis was carried out using IBM SPSS Statistics for Windows, Version 31 (IBM Corp., Armonk, NY). For continuous variables, the unpaired Student’s T-test and one-way ANOVA test were used to assess differences between two groups and three groups, respectively. Chi-square and Fisher’s exact test were used for comparisons of nominal variables. A p-value < 0.05 was considered statistically significant. Linear associations were evaluated using the Pearson rank correlation coefficient (*r*). Correlation strength was interpreted as follows: weak (0.21–0.40), moderate (0.41–0.60), strong (0.61–0.80), and very strong (0.81–1.00).

## Results

Between January 2022 and August 2024, 40 pediatric patients with NS and 40 healthy controls were enrolled in the study. The NS patients, whose mean age was 10.93 ± 2.78 years, were further subclassified into SSNS (15; 37.5%) and SRNS (25; 62.5%). The demographic characteristics and clinical data of the subjects included are presented in Table 1.

Table 2 presents a comparative analysis of conventional and tissue Doppler-derived RV functional parameters across the three study groups. TR was significantly more encountered in NS cases (trivial to mild degree), especially in SRNS, than in SSNS.

Tissue Doppler analysis revealed substantial differences statistically among groups regarding s’, e’, E/e’, and Tei index, with significantly lower s’, e’ in NS patients compared to controls, especially lower in SRNS compared to SSNS. Moreover, significantly higher E/e’ was detected in the NS group compared to controls, suggesting increased right ventricular filling pressures with more increased in SRNS than SSNS. The significantly higher Tei index in primary NS, particularly SRNS compared to SSNS, suggests global RV functional alterations impacting both systolic and diastolic components.

Table 3 provides a comprehensive comparison of RV deformation parameters derived from 2D-STE across the three study groups. RVFWLS and RV4CSL were significantly reduced in patients with NS compared with the healthy group, despite being within normal strain limits, indicating subtle alterations in myocardial mechanics. Segmental analysis using Auto Strain RV revealed significantly lower strain values in NS across the basal, mid, and apical segments compared to controls, highlighting region-specific variations in systolic RV functions.

Table 4 compares the study groups on 3D auto-RV-derived data. The mean 3DE-derived EF and SV were significantly lower in NS than in healthy subjects, with no significant difference between SRNS and SSNS. The comparison of auto-strain RV (Segmental) parameters showed varying trends across the segments (Base, Mid, Apical), with significant differences observed. Although TAPSE values were within the normal range for all groups, they were significantly lower in NS than in controls, especially in SRNS, compared with healthy subjects. For 3D-derived strains, mean values across all groups were not abnormal; however, the NS group had a lower free wall longitudinal strain than controls, and SSNS had a significantly lower free wall longitudinal strain than SRNS, with non-significant differences between the two groups in septal longitudinal strain.

Figures 1 and 2 illustrate the distribution of the mean values of different ECHO RV functional parameters across the three study groups.

In Table 5, Correlations are demonstrated between ECHO parameters in NS patients and clinical, laboratory, and therapeutic data. None of the Echo parameters studied correlated significantly with disease or therapy duration or relapse frequency. However, a significant negative correlation was detected with initial serum creatinine with 2D global strains of free wall and septum, and RV s’, last creatinine was correlated negatively with 3D derived RV EF. Similarly, negative correlations were detected between the last cholesterol level and s’ and 2D global longitudinal strains, but a positive correlation with the Tei index. Positive correlations were detected between the initial and last albumin with RV s’ and free wall longitudinal strain.

## Discussion

This study assessed RV function in children with primary NS, including both SSNS and SRNS, compared with controls using multiple echocardiographic functional modalities. This study represents a complementary analysis of the same cohort previously reported by our group, analyzing left ventricular function assessment [6].

The key finding was that children with primary NS exhibited significant RV functional alterations across multiple echocardiographic parameters, with significant correlations of some RV systolic functions with laboratory data but not with therapeutic or disease duration.

In literature, few studies have examined RV function using conventional or tissue Doppler imaging. Significantly lower TV E and E/A ratio and significantly higher myocardial performance index in our primary NS cohort compared to controls were consistent with Sadeghi-Bojd’s study, but with detected correlations with laboratory results [13]. Similarly, Saleh et al. found a significantly higher Tei index and lower E/A ratio in cases than in controls, although they found that the E/A ratio of the RV had a weak negative correlation and the Tei index had a moderate positive correlation with disease duration; a finding that was not supported by our data [14].

In our cohort of patients, a higher prevalence of trivial to mild TR was notable in NS cases, especially SRNS, with 2 cases of TR exceeding 2.8 m/s, suggesting the possibility of subtle, early developing pulmonary hypertension. Two other older studies showed that TR is more frequent in NS than in controls with higher pulmonary artery systolic pressure, although the prevalence was higher in their cohorts [15, 16]. A suggested explanation is pulmonary microthrombi secondary to the hypercoagulable state that is associated with this disease. Large pulmonary embolism is more documented in adults than in children with NS, but childhood pulmonary thromboembolism was reported [17].

The Tei index was significantly elevated in SRNS compared to SSNS and the control groups, suggesting global RV dysfunction in SRNS. The Tei index reflects both systolic and diastolic dysfunction and is considered a sensitive indicator of early RV impairment [18]. In the current study, TDI-derived e’, s’ were significantly lower and E/e’ was significantly higher in NS compared to healthy subjects, particularly the SRNS group, indicating impaired myocardial systolic function and impaired relaxation, consistent with diastolic dysfunction.

Children with SSNS showed a mild reduction in RV systolic and diastolic indices compared to controls, but these differences did not reach pathological thresholds. This may reflect transient or reversible myocardial effects related to short-term steroid exposure or minor hemodynamic shifts due to hypoalbuminemia and altered fluid status, which tend to normalize with remission. However, the consistent differences observed between SSNS and SRNS patients suggest a pathophysiological continuum of cardiovascular involvement, with SRNS representing a more advanced state of subclinical myocardial stress. This can be related to higher doses and prolonged exposure to steroid therapy in SRNS. Long term steroid use adversely affects myocardial function as it promotes myocardial hypertrophy, interstitial fibrosis, oxidative stress, and metabolic dysregulation, leading to impaired ventricular relaxation and subtle reductions in myocardial contractility before conventional echocardiographic indices become affected. The magnitude of affection varies according to the dose, duration, age of exposure and the underlying disease. Current evidence indicates that overt steroid-induced cardiomyopathy in older children is uncommon; however, prolonged glucocorticoid exposure may contribute to adverse myocardial remodeling and increase long-term cardiovascular risk through hypertension, insulin resistance, dyslipidemia, and obesity [19].

The more pronounced impairment in the SRNS group is multifactorial. It can be furthermore explained by disease severity, chronic systemic inflammation, hemodynamic factors, blood pressure, body composition, persistent proteinuria, differences in treatment, and exposure to nephrotoxic medications such as calcineurin inhibitors or alkylating agents, which have been associated with endothelial and myocardial injury [20, 21].

On using 2D-STE, there was a significantly impaired RVFWLS and RV4CS in patients with primary NS compared to healthy controls, although the mean strain values were within the normal average. The degree of strain impairment was most pronounced in the SSNS group for free wall strain but was not significant for RV4CS, indicating early subclinical systolic dysfunction. These findings are consistent with previous reports demonstrating that RV longitudinal strain is more sensitive for detecting subtle changes in myocardial contractility, particularly in pediatric populations with chronic systemic disease [9, 22, 23].

The reduced strain in primary NS cases compared to controls likely reflects the combined effects of chronic volume overload, systemic inflammation, and potential mitochondrial dysfunction or direct cardiotoxicity from prolonged immunosuppressive therapy. Segmental analysis further highlighted region-specific variations, with basal segments being most affected, suggesting non-uniform myocardial involvement. Such segmental heterogeneity may serve as an early indicator of regional myocardial remodeling in response to sustained disease burden [24]. Although basal right ventricular free-wall strain was significantly higher in the SRNS group than in the SSNS group, this isolated finding should be interpreted with caution. Regional right ventricular strain demonstrates substantial physiological variability owing to the complex anatomy and contraction pattern of the right ventricle. Furthermore, segmental strain measurements are more susceptible to tracking variability and sampling error than global strain parameters. Because this difference was not supported by corresponding improvements in other right ventricular segments, global RV free-wall strain, or conventional echocardiographic indices, it is unlikely to reflect genuinely preserved basal myocardial function. Instead, it may represent regional physiological heterogeneity or statistical variability related to the relatively modest sample size.

Regarding 3D auto-RV-derived parameters, they provided additional insights into RV performance. Our findings demonstrated a significant reduction in TAPSE, 3D ECHO-derived RV EF, SV, and 3D-derived strains in primary NS compared with the control group, with non-significant differences between SRNS and SSNS, except for RV free wall strain. These volumetric and functional changes confirm the presence of global systolic impairment. One theory is that cardiac edema associated with NS increases myocardial stiffness and subsequently affects contractility, which could be linked to aquaporins [25, 26].

Also, these results support the notion that 3D echocardiography offers a more comprehensive and geometrically accurate evaluation of RV function, especially in conditions where the RV undergoes subtle remodeling, which could not be accurately determined using 2D-derived functions [27, 28]. Furthermore, the automated quantification tools used in our analysis reduced observer variability and enhanced reproducibility, reinforcing the feasibility of using 3D echocardiography in clinical and research settings for early detection of cardiac dysfunction in high-risk populations [9, 29].

RV s’, Tei index, 2D-STE global strains, and 3D EF were correlated linearly with some of the laboratory data initially or at last follow-up, particularly serum creatinine, cholesterol, and albumin. This could indicate that the risk of cardiovascular changes may be linked to hypoalbuminemia, hyperlipidemia, and activation of the inflammatory cascade due to endothelial dysfunction, release of inflammatory cytokines, oxidative stress, and pulmonary bed thromboembolism [26, 30–33]. Consistently, Meshram et al. detected an inverse correlation of the Tei index of RV with serum albumin [33].

Taken together, the study’s findings underscore the progressive, subtle cardiomyopathic alterations that may develop even in the absence of overt clinical signs. Therefore, the integrative use of advanced echocardiographic modalities could serve as valuable tools for longitudinal monitoring of RV cardiac function in children with primary NS, especially those with treatment-resistant diseases.

### Strengths and limitations

The primary strength of this study is the use of a comprehensive echocardiographic approach to assess RV functions in a pediatric cohort with primary NS. Notably, this is the first study to apply both three-dimensional echocardiography and myocardial strain imaging for an in-depth evaluation of RV function in children with SSNS and SRNS.

However, the main drawback was the single-center design with relatively small sample size, particularly in the SSNS group, which may affect the generalizability of the findings. Moreover, long-term follow-up is needed to assess whether the observed RV dysfunction persists, progresses, or improves with changes in treatment or disease control.

### Clinical implications and future directions

Our findings highlight the need for regular cardiovascular surveillance in children with primary NS. This could help tailor therapies and possibly initiate cardioprotective interventions.

Further longitudinal studies with larger sample sizes and the incorporation of advanced imaging modalities, such as cardiac magnetic resonance (CMR), are warranted to validate these findings and explore their long-term clinical significance.

## Conclusions

Primary NS can subclinically affect RV cardiac function. A full understanding of RV status is possible through comprehensive use of echocardiographic modalities, including conventional, Tissue Doppler, 2D-STE, and 3D auto-RV. This data emphasizes the need for regular RV assessment of primary NS children. Future research on CMR and cardioprotective agents is warranted.

## Data Availability

The datasets used and/or analysed during the current study are available from the corresponding author on reasonable request.

## Abbreviations

ASE: American Society of Echocardiography
BMI: Body mass index
CMR: Cardiac magnetic resonance
ECHO: Echocardiography
EF: Ejection fraction
EDV: End-diastolic volume
ESV: End-systolic volume
GLS: Global longitudinal strain
ISKDC: International Study for Kidney Disease in Childhood
RV: Right ventricle
RVFWLS: RV free wall strain longitudinal strain
RV4CSL: RV four chamber longitudinal strain
RVFAC: Right ventricular fractional area change
STE: Speckle-tracking echo
SV: Stoke volume
NS: Nephrotic syndrome
SRNS: Steroid-resistant nephrotic syndrome
SSNS: Steroid-sensitive nephrotic syndrome
TDI: Tissue doppler imaging
TTE: Transthoracic echocardiograms
TAPSE: Tricuspid annular plane systolic excursion
2D: Two-dimensional
3DE: Three-dimensional echocardiography

## References

[1] Kitsou K, Askiti V, Mitsioni A, Spoulou V. The immunopathogenesis of idiopathic nephrotic syndrome: a narrative review of the literature. Eur J Pediatr. 2022;181(4):1395–404. 10.1007/s00431-021-04357-9. 35098401

[2] Hari P, Khandelwal P, Smoyer WE. Dyslipidemia and cardiovascular health in childhood nephrotic syndrome. Pediatr Nephrol. 2020;35(9):1601–19. 10.1007/s00467-019-04301-y. 31302760

[3] Trautmann A, Vivarelli M, Samuel S, Gipson D, Sinha A, Schaefer F, et al. IPNA clinical practice recommendations for the diagnosis and management of children with steroid-resistant nephrotic syndrome. Pediatr Nephrol. 2020;35(8):1529–61. 10.1007/s00467-020-04519-1. 32382828 PMC7316686

[4] Mahgoob MH, Setouhi AM. Subclinical systolic dysfunction in children with steroid-resistant nephrotic syndrome identified by speckle tracking echocardiography. BMC Pediatr. 2025;25(1):91. 10.1186/s12887-025-05449-3. 39905382 PMC11792370

[5] AbdelMassih A, Haroun M, Samir M, Younis S, Tamer M, Salem A. Hypoalbuminemia linked to myocardial dysfunction in recent-onset nephrotic syndrome: a cross-sectional case control 3DSTE study. Egyptian Pediatric Association Gazette. 2021; 69(1):24. 10.1186/s43054-021-00070-2.

[6] Hussein A, Rakha S, Hammad A, Hafez M, S Korkor M. Left ventricular myocardial functions in pediatric patients with primary nephrotic syndrome: a comprehensive evaluation using conventional echocardiography, two-dimensional speckle tracking, and three-dimensional echocardiography. Ital J Pediatr. 2025; 51(1):234. 10.1186/s13052-025-02086-5. PMC12261601 40660324

[7] Lang RM, Badano LP, Mor-Avi V, Afilalo J, Armstrong A, Ernande L, et al. Recommendations for Cardiac Chamber Quantification by Echocardiography in Adults: An Update from the American Society of Echocardiography and the European Association of Cardiovascular Imaging. J Am Soc Echocardiography [Internet]. 2015;28(1):1–e3914. 10.1016/j.echo.2014.10.003. 25559473

[8] Wang TKM, Grimm RA, Rodriguez LL, Collier P, Griffin BP, Popović ZB. Defining the reference range for right ventricular systolic strain by echocardiography in healthy subjects: A meta-analysis. PLoS ONE. 2021;16(8):e0256547. 10.1371/journal.pone.0256547. 34415965 PMC8378693

[9] Rakha S, Hammad A, Elmarsafawy H, Korkor MS, Eid R. A deeper look into the functions of right ventricle using three-dimensional echocardiography: the forgotten ventricle in children with systemic lupus erythematosus. Eur J Pediatr. 2023;182(6):2807–19. 10.1007/s00431-023-04936-y. 37039879 PMC10257604

[10] International Study of Kidney Disease in Children. Primary nephrotic syndrome in children: clinical significance of histopathologic variants of minimal change and of diffuse mesangial hypercellularity. A report of the International Study of Kidney Disease in Children. Kidney Int. 1981; 20(6):765-71. 10.1038/ki.1981.209 PMID: 7334749. 7334749

[11] chwartz GJ, Haycock GB, Spitzer A. Plasma creatinine and urea concentration in children: normal values for age and sex. J Pediatr. 1976; 88(5):828-30. 10.1016/s0022-3476(76)81125-0. 1271147

[12] Lopez L, Colan SD, Frommelt PC, Ensing GJ, Kendall K, Younoszai AK, et al. Recommendations for quantification methods during the performance of a pediatric echocardiogram: a report from the Pediatric Measurements Writing Group of the American Society of Echocardiography Pediatric and Congenital Heart Disease Council. J Am Soc Echocardiogr. 2010;23(5):465–95. 10.1016/j.echo.2010.03.019. 20451803

[13] Sadeghi-Bojd S, Noori NM, Teimouri A, Khaleghi A. Echocardiographic Findings in Children with Nephrotic Syndrome Compared with the Healthy Children. J Compr Ped. 2025;16(2):e161047. 10.5812/jcp-161047.

[14] Saleh SM, Elmaghraby KSM, Abdelfadil AM, Mohamed HS. Myocardial Performance Index in Nephrotic Syndrome. J Clin Exp Cardiolog. 2018;9:585. 10.4172/2155-9880.1000585.

[15] Rasslan L, Hassan B, Morsy S, Khalefa N. Assessment of the Pulmonary Artery Pressure in Children With the Nephrotic Syndrome. GEGET. 2007;7(1):1–12. 10.21608/geget.2007.31441.

[16] Du ZD, Cao L, Liang L, Chen D, Li ZZ. Increased pulmonary arterial pressure in children with nephrotic syndrome. Arch Dis Child. 2004;89(9):866–70. 10.1136/adc.2003.039289. 15321868 PMC1763209

[17] Ma F, Zhou K, Hua Y, Liu X, Duan H, Li Y, Wang C. Chronic thromboembolic pulmonary hypertension as the first manifestation of nephrotic syndrome in a 12-year-old child. Medicine (Baltimore). 2018; 97(41):e12349. 10.1097/MD.0000000000012349. PMC6203542 30313030

[18] Roberson DA, Cui W. Right ventricular Tei index in children: effect of method, age, body surface area, and heart rate. J Am Soc Echocardiogr. 2007; 20(6):764-770. 10.1016/j.echo.2006.11.002. 17543749

[19] Li W, Wang Y. Childhood systemic glucocorticoid exposure and subsequent alterations in myocardial structure and cardiac function: a narrative review of clinical evidence. Frontiers in Endocrinology. 2026 Mar 26;17:1781454. 10.3389/fendo.2026.1781454 PMC13061688 41970995

[20] Lovric S, Ashraf S, Tan W, Hildebrandt F. Genetic testing in steroid-resistant nephrotic syndrome: when and how? Nephrol Dial Transplant. 2016; 31(11):1802-1813. 10.1093/ndt/gfv355 PMC6367944 26507970

[21] Roberti I, Vyas S. Long-term outcome of children with steroid-resistant nephrotic syndrome treated with tacrolimus. Pediatr Nephrol. 2010; 25(6):1117-1124. 10.1007/s00467-010-1471-8. 20217433

[22] Pigatto E, Peluso D, Zanatta E, Polito P, Miatton P, Bourji K, Badano LP, Punzi L, Cozzi F. Evaluation of right ventricular function performed by 3D-echocardiography in scleroderma patients. Reumatismo. 2015;66(4):259–63. 10.4081/reumatismo.2014.773. 25829185

[23] Ladányi Z, Bárczi A, Fábián A, Ujvári A, Cseprekál O, Kis É, Reusz GS, Kovács A, Merkely B, Lakatos BK. Get to the heart of pediatric kidney transplant recipients: Evaluation of left- and right ventricular mechanics by three-dimensional echocardiography. Front Cardiovasc Med. 2023;10:1094765. 10.3389/fcvm.2023.1094765. 37008334 PMC10063872

[24] Wabich E, Dorniak K, Zienciuk-Krajka A, Nowak R, Raczak G, Daniłowicz-Szymanowicz L. Segmental longitudinal strain as the most accurate predictor of the patchy pattern late gadolinium enhancement in hypertrophic cardiomyopathy. J Cardiol. 2021; 77(5):475-481. 10.1016/j.jjcc.2020.11.004. 33246844

[25] Egan JR, Butler TL, Au CG, Tan YM, North KN, Winlaw DS. Myocardial water handling and the role of aquaporins. Biochim Biophys Acta. 2006;1758(8):1043-1052. 10.1016/j.bbamem.2006.05.021. 16876107

[26] Qin Q, Xu R, Dong J, Xia W, Sun R. Evaluation of right ventricle function in children with primary nephrotic syndrome. Pediatr Neonatol. 2010;51(3):166–71. 10.1016/S1875-9572(10)60031-9. 20675241

[27] Muraru D, Niero A, Rodriguez-Zanella H, Cherata D, Badano L. Three-dimensional speckle-tracking echocardiography: benefits and limitations of integrating myocardial mechanics with three-dimensional imaging. Cardiovasc Diagn Ther. 2018;8(1):101–17. 10.21037/cdt.2017.06.01. 29541615 PMC5835646

[28] Tolvaj M, Kovács A, Radu N, et al. Significant Disagreement Between Conventional Parameters and 3D Echocardiography-Derived Ejection Fraction in the Detection of Right Ventricular Systolic Dysfunction and Its Association With Outcomes. J Am Soc Echocardiogr. 2024;37(7):677–86. 10.1016/j.echo.2024.04.005. 38641069

[29] Posada-Martinez EL, Ivey-Miranda JB, Ortiz-Leon XA, et al. Association Between Three-Dimensional Right Ventricular Ejection Fraction and In-Hospital Outcomes in Patients Undergoing Cardiac Surgery: A Multicenter Study. J Am Soc Echocardiogr. 2025;38(8):685–93. 10.1016/j.echo.2025.02.008. 39984139

[30] Myette RL, Lamarche C, Odutayo A, Verdin N, Canney M. Cardiovascular Risk in Patients With Glomerular Disease: A Narrative Review of the Epidemiology, Mechanisms, Management, and Patient Priorities. Can J Kidney Health Dis. 2024;11:20543581241232472. 10.1177/20543581241232472. 38404647 PMC10894549

[31] Ashoor IF, Mansfield SA, O’Shaughnessy MM, Parekh RS, Zee J, Vasylyeva TL, et al. Prevalence of Cardiovascular Disease Risk Factors in Childhood Glomerular Diseases. J Am Heart Assoc. 2019;8(14):e012143. 10.1161/JAHA.119.012143. 31286821 PMC6662122

[32] Verma R, Deepthi B, Saha A, Bhattacharjee J. Endothelial Dysfunction in Children with Frequently Relapsing and Steroid-Dependent Nephrotic Syndrome. Indian J Nephrol. 2025;35(4):480–4. 10.25259/ijn_568_23. 40896612 PMC12392209

[33] Meshram RM, Mohite SM, Chopade S. Subclinical cardiac dysfunction in childhood nephrotic syndrome: a case–control ctudy. Heart Views: Off. J. Gulf Heart. 2025;26(2):108. 10.4103/heartviews.heartviews_51_25. PMC12571090 41170141

